# Abatacept for graft versus host disease prophylaxis in patients 60 years and older receiving mismatched unrelated donor transplantation for hematologic malignancies

**DOI:** 10.1038/s41409-023-02043-y

**Published:** 2023-08-14

**Authors:** Sharmila Raghunandan, Muna Qayed, Ben K. Watkins, Michael Graiser, Lev Gorfinkel, Adrianna Westbrook, Scott Gillespie, Brandi Bratrude, Aleksandra Petrovic, Yvonne Suessmuth, John Horan, Leslie S. Kean, Amelia A. Langston

**Affiliations:** 1 Aflac Cancer and Blood Disorders Center, Children’s Healthcare of Atlanta, Atlanta, GA USA; 2 Emory University, Atlanta, GA USA; 3 Winship Cancer Institute, Emory University, Atlanta, GA USA; 4 Dana-Farber Cancer Institute, Boston, MA USA; 5 Boston Children’s Hospital, Boston, MA USA; 6 Seattle Children’s Hospital, Seattle, WA USA

**Keywords:** Haematopoietic stem cells, Haematological cancer

## Introduction

Allogeneic hematopoietic cell transplant (HCT) remains the best option for a cure for many patients with hematologic malignancies, but is associated with significant morbidity and mortality, particularly for older patients. In the absence of a matched related or unrelated donor, a mismatched unrelated donor (MMUD) increases donor options, particularly for Black and Latinx patients. MMUD HCTs have historically been associated with an increased risk of severe acute graft-versus-host-disease (aGVHD), resulting in increased transplant related mortality (TRM) and decreased overall survival (OS) when compared with matched related or unrelated donor HCTs [1–4].

Abatacept, cytotoxic T-cell lymphocyte-4-immunoglobulin (CTLA4-Ig), is a T-cell co-stimulation blockade agent [5] that was granted FDA approval for the prevention of aGVHD in patients receiving unrelated donor HCT in part based on results of the *Abatacept 2* trial (ABA2, NCT01743131). ABA2 was a multicenter phase II trial that evaluated abatacept in addition to standard GVHD prophylaxis with a calcineurin inhibitor and methotrexate (CNI/MTX) for GVHD prevention in 8/8 HLA-matched unrelated donor (MUD) HCT and 7/8 MMUD HCT. Patients in the 7/8 MMUD cohort were assigned to a single arm open label stratum compared to a prespecified Center for International Blood and Marrow Transplant Research (CIBMTR) cohort and demonstrated a significant reduction in grade 3–4 aGVHD compared with the CIBMTR cohort receiving CNI/MTX alone. The lower rates of grade 3–4 aGVHD translated into significant improvement in TRM and OS at 2 years for patients receiving abatacept [6].

The ABA2 trial enrolled patients 6–76 years of age in the 7/8 MMUD analysis, but outcomes were not reported separately for patients over 60 years of age. This age group is at increased risk of TRM [7, 8] that is further compounded by a MMUD HCT and the epidemiology of myeloid malignancies. Therefore, the goal of this analysis was to evaluate outcomes in patients 60 years and older undergoing MMUD HCT for hematologic malignancies.

## Methods

We conducted a retrospective analysis of outcomes for patients 60 years and older who received a 7/8 MMUD HCT for hematologic malignancies and received GVHD prophylaxis with abatacept (4 doses, 10 mg/kg per dose, on days -1, +5, +14, and +28) + CNI/MTX between January 2015 and December 2021. The analysis included 9 patients who were enrolled on the ABA2 trial, and 13 subsequent consecutive “off-study” patients transplanted at Emory University using the addition of abatacept to CNI/MTX as part of standard of care GVHD prophylaxis.

Overall survival, disease-free survival (DFS), and the composite endpoint of “grade III–IV acute GVHD-free, severe chronic GVHD-free, relapse-free survival” (GRFS) are described as Kaplan-Meier survival probability estimates. Grade II–IV aGVHD, grade III–IV aGVHD, moderate to severe cGVHD, TRM and relapse are described as cumulative incidences estimates. These variables were estimated with competing risk analysis in all patients where death or relapse acted as a competing event. Patients contributed person-time until the date of the event, the competing event if applicable, or until their last follow-up, whichever occurred first. All estimates are accompanied by 95% confidence intervals (CI). Acute GVHD is analyzed at 180 days, while all other endpoints are analyzed at 1 year. Group comparisons included Transplant Comorbidity Index (HCT-CI) (<3 versus >=3) and disease risk index (DRI) (low and intermediate DRI vs high and very high DRI) and were conducted with Gray’s test for competing risk analyses and with Log-rank tests for Kaplan-Meier analyses. All analyses were conducted with the lifetest procedure and figures were generated with the newsurv macro in SAS 9.4 (Cary, NC).

## Results

Twenty-two patients were included in this analysis, 9 who were enrolled in the ABA2 trial and 13 who were “off-study” patients. Median age at HCT was 66 years (range 60-76 years). The most common indication for transplant was MDS followed by AML (68% and 23% respectively). 32% of patients had high or very high DRI and 50% of the patients had >=3 HCT-CI. 90% of patients received reduced intensity conditioning with fludarabine/melphalan and 10% received myeloablative conditioning with busulfan/cyclophosphamide. 86% of patients received peripheral blood stem cell grafts. 68% of donors were younger than 35 years. Median follow-up for survivors was 2 years.

**Fig. 1:**
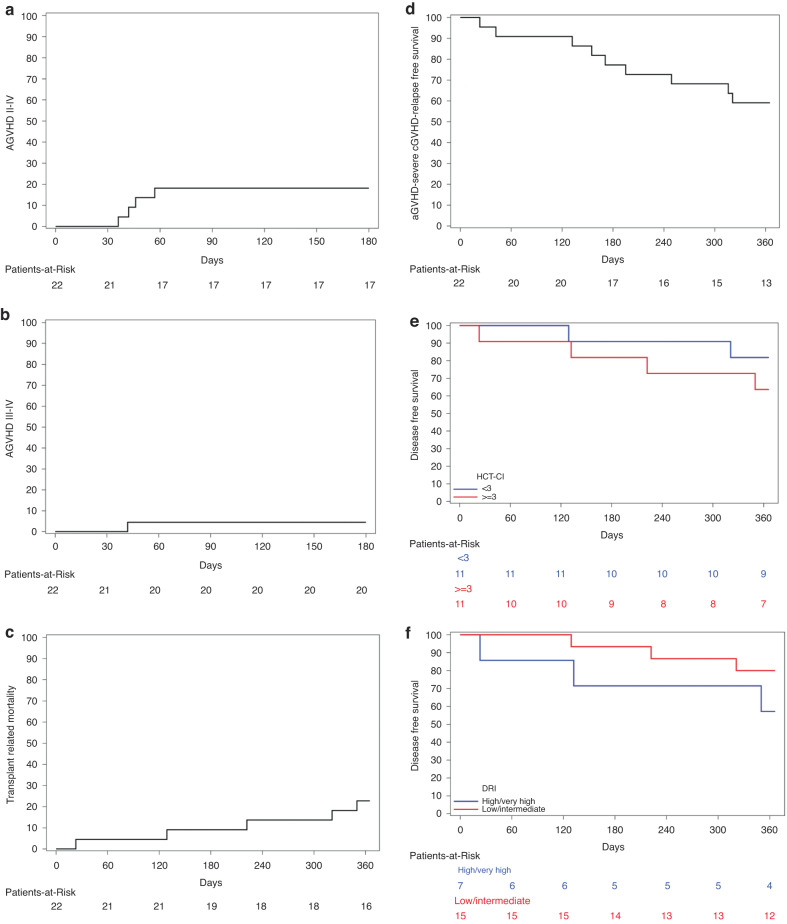
Cumulative incidence of GVHD and Kaplan-Meier plots of survival outcomes. **a** Cumulative incidence of Grade 2–4 aGVHD. **b** Cumulative incidence of Grade 3–4 aGVHD. **c** Cumulative incidence of Transplant Related Mortality. **d** Kaplan-Meier plot of Grade III–IV acute GVHD-free, severe chronic GVHD-free, elapse-free survival (**e**). Kaplan-Meier plot of Disease-Free Survival based on Transplant Comorbidity Index (HCT-CI) of <3 vs >=3. **f** Kaplan-Meier plot of Disease-Free Survival based on Disease Risk Index (DRI), low and intermediate DRI vs high and very high DRI.

There was one death prior to day 30, but all other patients achieved neutrophil engraftment by day 30 and platelet engraftment by day 100. The cumulative incidence of grade II–IV aGVHD by day 180 was 18% (95% confidence interval, CI, 6–37%, Fig. 1a) and the cumulative incidence of grade III–IV aGVHD by day 180 was 5% (95% CI 0.3–19%, Fig. 1b). One patient developed grade II late aGVHD at day +264. The cumulative incidence of moderate-severe chronic GVHD (cGVHD) at 1 year was 62% (95% CI 37–80%). The cumulative incidence of TRM and relapse at 1 year was 23% (95% CI 8–42%, Fig. 1c) and 5% (95% CI 0.3–20%) respectively, resulting in DFS of 73% (95% CI 49–87%), and OS of 77% (95% CI 54–90%). GRFS at 1 year was 59% (95% CI 36–76%, Fig. 1D). With respect to HCT-CI of <3 vs >=3, there were no statistically significant differences in TRM (18% vs 27%, *p* value = 0.62), OS (82% vs 73%, *p* = 0.62) and DFS (82% vs 64%, *p* = 0.35, Fig. 1e) between the two groups. There were also no statistically significant differences in TRM (20% vs 29%, *p* = 0.67), OS (80% vs 71%, *p* = 0.67) and DFS (80% vs 57%, *p* = 0.26%, Fig. 1f) between low and intermediate DRI vs high and very high DRI respectively.

## Conclusions

This study is the first to specifically report outcomes for patients 60 years and older receiving 7/8 MMUD transplants for hematologic malignancies and employing an abatacept-containing GVHD prophylaxis regimen. The results of this analysis reveal low rates of grade III–IV aGVHD and relapse and encouraging DFS in this population. These results are promising, particularly given the older age group and the fact that 32% of patients had high or very high DRI and 50% of the patients had >=3 HCT-CI. These results also confirm morbidity and mortality outcomes comparable to those described in the HLA-matched setting, while preserving the antitumor effect afforded by administration of relatively intensive conditioning [7, 8]. Similar to results from the ABA2 trial, cGVHD rates did not appear to be impacted with the 4 dose abatacept regimen, and an on-going study is evaluating extended dosing of abatacept to further improve cGVHD (ABA3, NCT04380740). There are some limitations of this analysis including its retrospective nature, the small sample size and the inclusion of 9 patients treated on the ABA2 trial. Larger and multi-center real-world studies will be needed to validate results from our analysis.

## Data Availability

Data collected for this study will be made available with publication to anyone who wishes to access the data for any purpose; please contact the corresponding author: sraghu2@emory.edu/sharmila.raghunandan@gmail.com.
